# Performance analysis of innovative cleaning and soiling mitigation solutions in the semi-arid climate of Benguerir Morocco

**DOI:** 10.1016/j.heliyon.2023.e16163

**Published:** 2023-05-11

**Authors:** Abderrazzak Elamim, Said Elhamaoui, Khalil Tijani, Aboubakr Benazzouz, Cesar Martins, Bruno Queiroz, Clara Faria, Abdellatif Ghennioui

**Affiliations:** a Green Energy Park, Regional Road Kelaa km^3^, R206, 43150, Benguerir, Morocco; b ChemiTek, Rua Dr. Francisco Sá Carneiro, Letra C, 4740-010, Esposende, Portugal; c IESI Laboratory, ENSET Mohammedia, Hassan II University of Casablanca, Morocco

**Keywords:** Photovoltaic, Outdoor exposition, PV Soiling, Anti-soiling coatings, PV Cleaning, Outdoor performance

## Abstract

The objective of this paper is to evaluate the performance of the hydrophobic coatings and detergent cleaning & antistatic protection for photovoltaic solar panels in semi-arid weather conditions in Benguerir Morocco. Various coating and cleaning strategies were tested on five photovoltaic (PV) systems with the same PV panels and electrical configurations. The first PV system (uncleaned) was not subject to any coatings or cleaning solutions. The second PV system (Water Cleaned) was periodically cleaned with raw water. The third PV system: solar wash protects (SWP) made use of a cleaning solution. The fourth:D solar defender (DSS) and fifth: industrial glass protect (IGP) PV systems each had a unique combination of the two hydrophobic coatings. The results demonstrated that after 9 months of operation, in the first 3 months (cleaning period) the average efficiency gain of the coated PV panels is around 10% compared to the reference system. Whereas in the non-cleaning period after 6 months of exposure, the efficiency gain is around 5%. After the outdoor exposure period, the cumulative energy gain of the coated systems compared to the water-cleaned reference reaches an average of 3%. It has been found that the SWP used 50% less water to clean the PV panels than the system cleaned without a cleaning solution, which made the panels harder to clean. The SWP is more successful at dust removal during the dry season (August–February) with low rain rates. However, during the rainy season (March–April), IGP outperformed SWP and DSD, with a small difference in PV performance. This study demonstrates the significance of using new cleaning strategies such as anti-soling coatings in dry areas to enhance the performance of photovoltaic systems, which may be useful for investors, researchers, and engineers interested in grid-connected photovoltaic and self-cleaning technology.

## 1 Introduction

The swift exhaustion of traditional energy resources. has led to an intensive search for new, more efficient, and green power plants with advanced technology. New clean energy and fuel technology are the subjects of extensive research and investigation currently, which coincides with an increase in environmental protection concerns on a global scale [1]. The proportion of renewable sources contributing to the generation of power around the globe increased to 29% in 2020, up from 27% in 2019 [2]. Solar power is an attractive option for the generation of electricity since the sun is a free, abundant, and environmentally friendly source of energy [3].

In the case of photovoltaic systems, many efforts have been made to improve the efficiency of PV modules in recent years. However, the actual field operating efficiency of PV modules is not properly predicted by the specifications, as they are associated with standard test conditions (STC) that are not compatible with actual field conditions [4].

Therefore, detailed knowledge of the prevailing weather and climate conditions at the sites where the PV systems operate is very important to obtain sufficient information about the behavior of the PV plants and their operational efficiency [5].

Nonetheless, there are many variables that can influence the module performance and lifetime (e.g. solar intensity, ambient temperature, wind speed, and high UV doses), especially in arid and semi-arid climates [6,7]. In addition to all these factors, dust is another factor that causes a significant drop in solar energy production and can lead to problems with durability and performance such as delamination, discoloration, hot spots, and power loss [8,9]. Dust accumulation is an important issue in PV systems that needs to be considered; it can gradually degrade the efficiency in the order of 1%–6.2% [10]. Consequently, a large number of researchers are interested in determining the amount of electrical energy that is lost because of the effect of soiling. According to the study that was presented in articles that have been previously defined [11], soiling can be transported through several different mechanisms, depending on wind speed and particle size. These mechanisms include creeping, saltation, short-term suspension, and long-term suspension. In fact, the creeping process refers to particles of soiling with a diameter higher than 500 μm. Air humidity condenses on surfaces in open natural environments. It has been reported that humidity enhances particle adherence to surfaces and the wind force needed to resuspend particles [12]. In the desert and semi-arid areas, dust particles with a diameter between 2 and 63 μm cause most soiling, since larger sand particles (>63 μm) are too heavy to settle on PV modules unless during desert storms [13].

Today's trend is to keep or improve the performance of photovoltaic (PV) modules, and this can be done using tracking systems as well as cleaning methods that are both inexpensive and friendly to the environment. Several cleaning systems have been developed with the intention of preventing significant losses of power generated by solar panels because of soiling. These losses of power can be mitigated by an operation and maintenance strategy (i.e. cleaning strategy), which has led to the development of these cleaning systems [14].

In Morocco, Hajjaj et al. examined the performance drop of a photovoltaic system composed of a new PV module conception without EVA encapsulation after three years of operation under harsh conditions. The system experienced an energy loss of 1.8 kWh in one string due to cracks and breaks in the module cells caused by bad manual cleaning and the absence of EVA protection. The researchers recommend the use of PV modules with EVA encapsulation to protect against breakage or mechanical stress in regions with high soiling rates and frequent cleaning events [15]. Ammari et al. studied the impact of soiling on the temperature and electricity production of two photovoltaic technologies (Poly-Si and CdTe) under a hot semi-arid climate of Morocco, based on a one-year experiment in which two modules from each technology were exposed in outdoor conditions, with one module cleaned daily and the other left to accumulate soiling. The study measured the daily soiling ratio (SR) and its impact on electricity production and module temperature (Tm). Results showed that Poly-Si modules were more affected by soiling than CdTe modules, with daily energy production drops of 15% and 13% respectively. The authors also highlighted the importance of regular cleaning and proposed a model to predict the impact of dust deposition on module temperature based on the SR measurements [16]. The impact of soiling on photovoltaic (PV) and concentrated solar power (CSP) technologies in a semi-arid climate in Morocco was investigated by Abraim et al. The study utilized data obtained from soiling sensors to determine that CSP exhibits a significantly higher optical soiling loss compared to PV. The researchers also found that the total annual energy loss attributed to soiling is 17.76% and 1.95% for CSP and PV, respectively, when a monthly cleaning frequency is considered. The study conducted by the researchers has revealed that the most effective cleaning frequency for PV and CSP solar plants is once every three weeks. They have suggested the incorporation of soiling sensors in all site assessment and pre-feasibility projects, as well as in operational solar power plants for performance monitoring. Additionally, the researchers have recommended the consideration of soiling as a dynamic parameter [17].

In recent years, one of the topics that have garnered a lot of attention is the implementation of mitigation and cleaning procedures. Various research studies have resulted in the development of different cleaning strategies. These methods and techniques are costly, depending on the type of cleaning system. These include natural, manual, and automatic cleaning methods [18]. Natural cleaning depends on rainfall; hence, hand cleaning is necessary for dusty regions. Given labor expenses, corrective and preventive cleaning categories were created. Corrective cleaning includes human cleaning, mechanical approaches that can be fully automated or semi-automated, and the electrodynamic screen. Preventive cleaning involves treating the solar panel surface [19]. For the manual cleaning technique, human resources, equipment such as dry cleaning, as well as water in the case of wet cleaning, are needed to clean the front surface of the PV module. However, it is expensive, there is limited water in arid regions, and scratches might occur [20].

Mechanical cleaning systems refer to any cleaning procedure that uses motorization to replace the operator's physical labor. They are distinguished by their powerful dust removal capacity, rapid operation, environmental adaptability, and control performance [21]. The National Aeronautics and Space Administration (NASA) uses the electrodynamic screen (EDS) as the main way to get rid of the dust on Mars and Moon missions. This method has been suggested and tested in different environments [22]. When compared to other procedures, EDS is viewed as being much faster. However, exposure to ultraviolet (UV) radiation poses a risk of screen damage [23]. In several cases, dust and dirt are not effectively removed by the cleaning method (nanometers). Furthermore, the interaction of certain of these techniques with specific contaminants may speed up the PV corrosion process [24]. Preventive soiling mitigation includes different approaches aiming to repel the dust from the panel's surface based on the treatment of the surface properties [21]. This category of soiling mitigation relies on painting the PV surface with a coating that can be hydrophobic or hydrophilic, functioning as a barrier and preventing water droplets from adhering to the panel.

This form of coating prevents the dust from adhering to the PV surface; however, it requires the use of water to remove the dust [25]. Water collection rates are improved by 95% when coatings are applied to the PV module's surface, in comparison to an uncoated glass surface, and by 51% when compared to uniformly coated low-iron glass [3,26]. Many articles exist on the impact of dust accumulation on the PV module surface. Several studies have examined the impact of utilizing hydrophobic nano-coatings and cleaning solutions on the efficiency of photovoltaic (PV) systems. The utilization of an antistatic coating in conjunction with a mechanical vibrator has demonstrated a successful reduction in the frequency of cleaning required for monocrystalline PV panels. Specifically, the application of a coating coupled with a vibrating system has resulted in a decrease in the required cleaning frequency from 4 times per month for non-coated panels to 2 times per month for coated panels. This finding suggests that dust mitigation through the use of panel coatings is an effective technique for improving the performance of PV panels. However, if a vibrating system is not applied, an inclination study should be conducted [27].

In [28], applying a hydrophobic coating film under dew suppression by covering the samples from sunset to sunrise in a semi-arid climate zone reduces particles deposition by 60.0 ± 8.1% compared to the uncoated slides (under dew suppression) and reduces the % of soiling loss (50 ± 13%) compared to the coated slides without dew suppression (31 ± 15%), however this study does not address on a better understanding of the impact of anti-soiling coatings on Dust Potency DP, more data was required to have. Whereas Benjamin [29] summarize recent PV machine-cleaning arc's abrasion studies, with a focus on comparing techniques and conditions between laboratory and real field conditions, portable reflectometers are useful to characterize the antireflective coating of PV modules in the field, and for consistent results one should position the sensor between metal busbars of PV cells and use data from midrange wavelengths, light microscopy is often used in conjunction with reflectometers and lab profilometers to assess the visual appearance of scratches. It is important to note that the existing literature covers only environmental factors, generic dust deposition methods, and classified cleaning solutions. compared to the above-mentioned review articles, The study conducted by Khalid et al. [10] investigates the factors of dust accumulation and aggregation on photovoltaic (PV) panels. The paper additionally examines the mathematical modeling of photovoltaic panels in relation to their susceptibility to dust accumulation [10].

As far as the authors are aware, no article has been produced that encompasses a comprehensive survey of the effects of dust, its analysis, mathematical modeling, and potential methods for mitigating dust deposition. In their study, Khalid et al. [10] provide an overview of various photovoltaic (PV) panel cleaning techniques, including electrostatic, robot cleaning, self-cleaning nano films, automated, semi-automated, robotic, and unmanned aerial vehicle (UAV)-based dust cleaning methods. The objective of this article is to conduct a review of a cleaning solution that is sustainable in an environment characterized by the presence of dust, This article also aims to review a sustainable cleaning solution in a dusty environment. Many articles are present that performed the investigations of the PV system based on different sites. Still, few reports have considered the effect of cleaning techniques using hydrophobic coating on SPV productivity. Therefore, to fill the current research gap, the performance analysis and evaluation of performance indices for different PV systems (uncleaned PV system, Water Cleaned PV system, solar wash protect (SWP), D solar defender (DSD) and industrial glass protect (IGP) PV systems each had a unique combination of two hydrophobic coatings) are also considered in this article which is the novel contribution of the present research.

This research provides an analysis of the application of two novel hydrophobic coating and cleaning solutions on an 11.725-kWp mono-crystalline PV panel, based on experiments in real exposure conditions. The tests were performed in the climatic conditions of the city of Ben Guerir located in Morocco characterized by a semi-arid climate. The findings of this study will also be of interest to solar project developers, investors, and decision-makers to more correctly assess the losses caused by soiling and cleaning operations, as well as to enhance the management of their solar systems.

## 2 Experimental setup and procedure

### 2.1 Overview of the grid-connected PV system

**Fig. 1:**
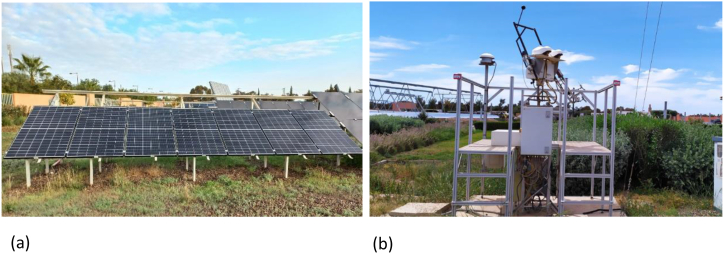
(a) PV system installed; (b) High precision meteorological station.

**Fig. 2:**
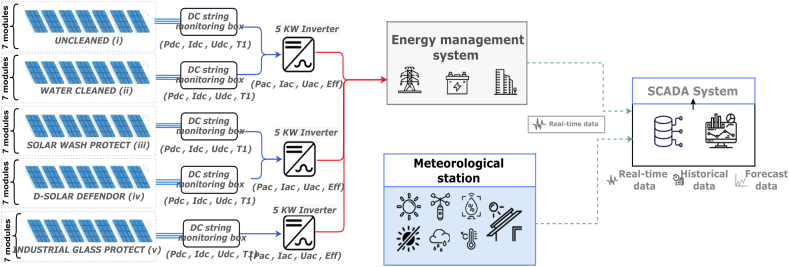
Outdoor test field monitoring architecture.

The outdoor test is carried out on five monocrystalline (mc-Si) PV systems with a total rated power of 11.725 kWp (2.345 kWp each) using up-to-date industry technology (Half-cells PERC), each PV string is formed by 7 modules in series (330 Wp each). The PV panels are installed in the research platform Green Energy Park (GEP) in Benguerir, Morocco, at an elevation of 449 m above sea level and latitude of 32.2° N, and longitude of 7.92° E. The unshaded fixed panels were mounted and tilted at 32° to the true south. Each PV system has an inverter with a rated capacity of 5000 W and a rated maximum efficiency of 97%. The technical specification of the solar photovoltaic panels and inverter are described in Table 1. The monitoring system was set to measure electrical and meteorological parameters. Electrical data (Pdc, Pac, Uac, Udc, Idc, Iac, energy yields, inverter temperature, string currents, module temperature) are collected using a DC monitoring device and the Inverter using a 1-min time step, while the meteorological data (global, direct, diffuse & in plane of array irradiances, Ambient temperature, Wind speed & direction, Relative humidity, Rainfall & duration) are collected via the meteorological station (presented in Fig. 1b) with a 10 s time stamps precision. Fig. 1 a shows a picture of the studied PV systems installed at GEP and system archetype is shown in Fig. 2.

**Table 1:** PV system characteristics.


***PV module***	Module technology	PERC, Mono-Si
	Pmax	335 Wp
	Impp	9.85 A
	Vmpp	34.0 V
	Isc	10.48 A
	Voc	40.7 V
***PV string***	Installed PV power	11.72 KWp
	Installed power per string	2.34 KWp
	Module tilt and orientation	32°, South
	Nbr. of modules per string	7
	Nbr. of strings	5
	Installation date	August 2021
***Inverter***	Max PV power	5.52 KW
	Max input voltage	600 V
	Max. input current per MPPT	11 A
	Max. efficiency	98.6%

The PV modules are cleaned periodically with tap water and a brush, with details of cleaning.

**Fig. 3:**
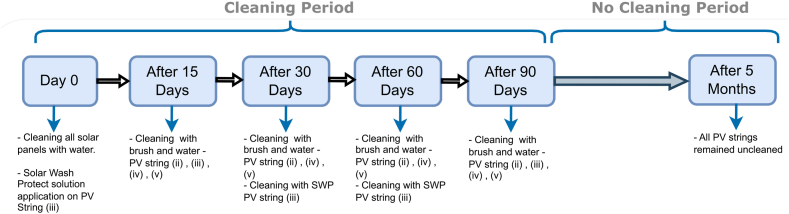
PV systems cleaning process protocol.

Described in Fig. 3 below, the period of the test is divided into two main parts: The cleaning period lasts for 3 months and was done during the dry season between the period of August 2021 and November 2021. No Cleaning period comes as a continuity for the previous period by keeping all systems under natural cleaning as this period represents the rainy season between November 2021 and April 2022.

### 2.2 Nano-particles coatings preparation and application

This study proposes three cleaning methods, which are discussed below.

#### 2.2.1 Solar wash protect (SWP)

The SWP concentrated solution was made by adding individual raw materials (the surfactants, the polymer, the preservative, and the dispersing agents) to water and mixed for 10–20 min, at room temperature, until a homogeneous solution of pH between 6 and 8 was obtained.

For the application, the SWP concentrated product was diluted in water (in a 1:50 ratio), to create ready-to-use SWP washing solution. The SWP washing solution was sprayed directly to the solar panel and a brush was used to scrub the entire surface of the panel. After that, the solar panel was rinsed with water to reduce any dispersed dirt or excess product left on the surface, and the panel was left to dry on its own.

#### 2.2.2 Industrial glass protect (IGP)

To produce the IGP hydrophobic coating, the silicone polymers and the solvent were added to water as individual raw materials and mixed for 20–30 min, at room temperature, until a homogeneous solution of pH between 4 and 4,5 was obtained. The surface of the solar panel was cleaned and degreased using alcohol and a microfiber cloth. Latex gloves were used throughout the entire process to ensure a completely clean and dry surface. A fine mist of IGP was applied to the surface, spraying the solution between 30 and 40 cm (the recommended volume is approximately 5–10 mL/m2). The solar panel's surface was immediately polished with a microfiber cloth and left to dry, for at least 1.5–2 h without any contact with water or any other liquids.

#### 2.2.3 D-solar defender (DSD)

To produce the DSD concentrated hydrophobic coating, the surfactant and preservative agent were added to the main polymer in its composition as individual raw materials and mixed for 20–30 min, at room temperature, until a homogeneous solution of pH between 6 and 8 was obtained. Related to the application of the product to PV modules, the DSD concentrated product was diluted directly in distilled water, in a 1:1000 dilution ratio. The DSD ready-to-use solution was applied to the solar panel and a soft brushed was used to scrub the glass. After this, the panel was left to dry on its own.

NB: The hydrophobic coatings and the Solar Wash Protect are developed and applied by ChemiTek company, Esposende, Portugal.

### 2.3 Experimental procedure

An experimental setup has been developed to evaluate the performance of the hydrophobic coatings and detergent cleaning & antistatic protection and their sensitivity to soiling in hot climatic conditions as well as the possible degradation mechanisms, an outdoor testing exposure using five identical PV systems was performed.

1The first system without any type of coating or cleaning protection is used as a reference for comparison.
2The second system “Water Cleaned” is the cleaning with only water, without any type of coating.
3The third system uses a Solar Wash Protect (SWP), a concentrated product developed to clean solar panels. SWP is a water-based product, with different surfactants and dispersing agents in its composition, that are responsible for removing the dirt with great efficiency, as well as polymer with antistatic properties, that adhere to the surface glass after the cleaning process.
4The fourth system is coated with a D-Solar Defender (DSD), which is a highly concentrated hydrophobic coating; DSD is a solvent-free product, based on polymer with hydrophobic properties that adheres to the glass surface of the solar panels.
5The fifth system is coated with an Industrial Glass Protect (IGP), is a long-lasting hydrophobic coating, IGP has polymer in its composition, responsible for its hydrophobic properties, but it is free of fluorocarbons, TiO2 and other dangerous compounds for the environment. Unlike DSD, IGP does not require dilution and it lasts for longer periods of time (IGP lasts for up to 5 years, unlike DSD only lasts up to 1 year);

The hydrophobic coatings and the Solar Wash Protect are developed and applied by ChemiTek company, Esposende, Portugal. The system has been evaluated during the dry season (August 2021–November 2021) and the rainy season (December 2021–April 2022) in Morocco at the Green Energy Park located in Ben Guerir. During this time, the electrical parameters of the PV system are collected by the monitoring system along with the weather parameters collected by the meteorological station within the same site location.

### 2.4 Performance assessment parameters

#### 2.4.1 Electrical performance measurement

Current-voltage (I–V) and power voltage (P–V) curve scanning are two primary methods often used by researchers to monitor and assess a module's electrical performance. These measurements demonstrate the maximum power output (Pmax), maximum output current (Imax), maximum output voltage (Vmax), open circuit voltage (Voc), and short circuit current values of a module (Isc). The electrical properties of the system and individual module samples were examined using outdoor RTC I–V curve measurements to further assess the difference between the installed systems. The PVPM1040CX I–V curve tracer was employed in this inquiry to assess the system under real test conditions (RTC). The I–V measurement test is conducted outdoors after each cleaning cycle (day 0, day 15, day 30, day 60). The cleaning of modules beforehand minimizes the impact of soiling on the electrical parameters of the system and each measurement is replicated 3 times to avoid sensor inaccuracy.

To perform comparative measurements under various irradiance and temperature settings, the IV tracer normalizes recorded values in Real Time conditions (RTC) to Standard Test Conditions (STC 1000 W/m^2^, 25 °C) following conversion equations Eq. (1) & Eq. (2) in accordance with IEC 60891 standards [30]:

(1) $$I=I_{1}+I_{SC1}\;\left(\frac{G}{G1}-1\right)+\alpha \;\left(T-T_{1}\right)$$

(2) $$V=V_{1}-R_{s}\left(I-I_{1}\right)-\kappa *I*\;\left(T-T_{1}\right)+\beta *\;\left(T-T_{1}\right)$$

where: I_1_.V_1_ are respectively the measured current and voltage value, I, V are respectively corrected current and voltage value at STC, G1 is the irradiance measured with the reference cell, G is the irradiance at STC, T1 is the temperature of the test specimen and T_2_ is the cell temperature at STC, α and β are current and voltage temperature coefficient, Rs is the internal series resistance of the test specimen and κ is the curve correction factor. To minimize the effects of irradiance and temperature fluctuations on STC conversions, measurements are taken during peak hour irradiance (12 a.m.) with optimal clear sky conditions. The maximum measurements uncertainty ranges between ±3% and ±5% for module temperature and irradiance respectively.

#### 2.4.2 Performance analysis of SPV modules

Multiple KPIs have been set to analyze the behavior of the coating and cleaning solution with respect to the cleaning protocol and the variation of meteorological conditions. Below defined the indicator of performance used for the analysis following the IEC 61724 norms to perform the PV analysis and evaluation [31].

##### 2.4.2.1 Performance ratio

The PR indicates the relationship between actual and desired yield. It denotes PV module losses caused by heat, soiling, deterioration, or operational issues [31]. The difference between the array yield and the reference yield is represented by the DC performance ratio. It is a critical performance indicator for analyzing the behavior of the PV system while just considering its DC side, allowing us to distinguish between PV strings without the effect of any other component (inverter, BOS, etc.). The following Eq. (3) calculates the performance ratio:

(3) $$\mathrm{PR}=\frac{\frac{Output\;energy}{Power\;at\;STC}}{\frac{Irradiance}{Irradiance\;at\;STC}}=\frac{Y_{A}}{Y_{R}}\;\left(\frac{KWh}{KWp}\right)$$

##### 2.4.2.2 Array yield

The array yield refers to the amount of DC energy produced by a photovoltaic (PV) system over a specific period of time, which is then divided by the system's rated power to normalize the result. Additionally, it indicates the number of hours during which the PV system operates at its rated capacity [32,32]. The Y_A_ is calculated using Eq. (4) below:

(4) $$Y_{A}=\frac{E_{DC}}{P_{PV\;rated}}\;\left(\frac{KWh}{KWp}\right)$$

where: $E_{DC}$: is the DC energy in kWh; $P_{PV\;rated}$: is the installed Capacity (kW).

##### 2.4.2.3 Capture losses

1DC capture losses of PV panels refer to the amount of energy lost in the conversion of sunlight into DC electricity due to factors such as shading, temperature, and wiring resistance. Eq. (5) below presents the calculation formula:

(5) $$L_{C}=Y_{R}-Y_{A}\;\left(\frac{KWh}{KWp}\right)$$

With: $Y_{R}$ Is the reference yield; $Y_{A}$ is the Array yield;

##### 2.4.2.4 Cumulative energy gain

Represents the gain of energy for each system taking as a reference the water-cleaned system [33]. The following Eq. (6) calculates the performance ratio:

(6) $$Gain\;Energy=\left(\frac{Running\;Total\;\left(P_{sys}\right)}{Running\;Total\;\left(P_{WC}\right)}-1\right)*100$$

## 3 Results and discussion

### 3.1 Local site dust analysis

This part will be devoted to the analysis of the dust in the city of Benguerir To analyze the chemical and mineralogical characteristics of dust particles and to give an overview of the local dirt loss of each site. The collected dust samples were subjected to the analyses (Characterization Methods) described below.

1Physical and chemical properties were determined by X-ray fluorescence (XRF).
2Mineralogical analysis of the dust sample was performed using FTIR (Fourier Transform Infra-Red spectroscopy).
3The surface morphology is studied by scanning electron microscopy (SEM).

The mineral analysis by X-ray fluorescence and the corresponding atomic weight percentage distribution of the dust samples is shown in Table 2.

**Table 2:** XRF mineral quantitative analysis.

Minerals	SiO_2_	TiO_2_	Al_2_O_3_	Fe_2_O_3_	MnO	MgO	CaO	Na_2_O	K_2_O	P_2_O_5_
Sample 1	20.68	0.25	3.30	0.40	0.02	3.75	36.21	1.20	0.38	15.85
Sample 2	24.33	0.24	3.60	0.72	0.02	3.35	31.11	2.70	0.34	19.65
Sample 3	31.83	0.4	6.70	1.49	0.03	2.33	25.55	2.40	0.74	14.51
Sample 4	22.32	0.27	4.64	0.82	0.02	4.38	32.43	0.56	0.57	18.45
Sample 5	17.11	0.18	8.89	0.49	0.02	3.93	36.47	1.08	0.34	23.01

The elements identified in the dust sample may be in the form of various minerals as presented in Table 2 and It was observed that the percentage of concentration of calcium oxide (CaO) varies between 36.47% and 25.55%, the concentration of quartzite (SiO_2_) varies between 31.83% and 17.11% of the content of dust particles followed by phosphorus (P_2_O_5_) with a concentration that varies from 23.01% to 15.85%, as well as other compounds with minor amounts such as: MgO, Na_2_O, K_2_O, TiO_2_, MnO, Al_2_O_3_, Fe_2_O_3_. The samples are analyzed by Fourier transform infrared spectrometry in transmission mode with a NicoletTMIsTM50 spectrometer.

**Fig. 4:**
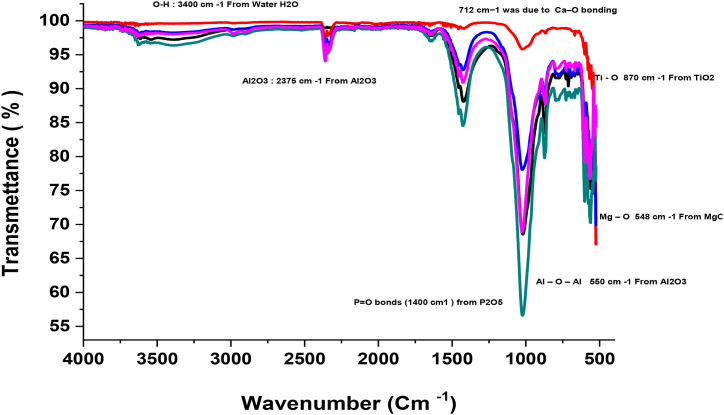
Infrared spectroscopy (FTIR) of dust sample.

Fig. 4 shows the FTIR analysis of the samples overlaid by infrared spectroscopy, indicating.

1The detection of aluminum oxide Al_2_O_3_: A band at 550 cm^−1^ and a band at 2375 cm-1characteristic of elongation and deformation vibrations of the Al–*O*–Al bond are significant in the presence of aluminum oxide.
2Quartz detection: A band at 1087 cm^−1^ characteristic of the Si–*O*–Si bond significant of the presence of silicon dioxide (Silica) in the samples
3The detection of the ferric oxide: A band at 587 cm^−1^ characteristics of the bond Fe–O significant of the presence of the iron oxide (III) in the samples
4The peak at 870 cm^−1^ represents the presence of titanium dioxide in the samples.
5The peak at 712 cm-^1^ indicates the presence of lime in the samples.
6The peak at 3400 cm^−1^ represents the presence of H_2_O
7The absorption peak at 1400 cm^−1^ indicates the presence of P_2_O_5_ (phosphorus) in the samples.
8The absorption peak of 548 cm-1 indicates the presence of Magnesium Oxide.

Calcite, dolomite, phosphorus and quartz are the major components of the local dust, as a result of the industrial activities in the area and the construction work.

**Fig. 5:**
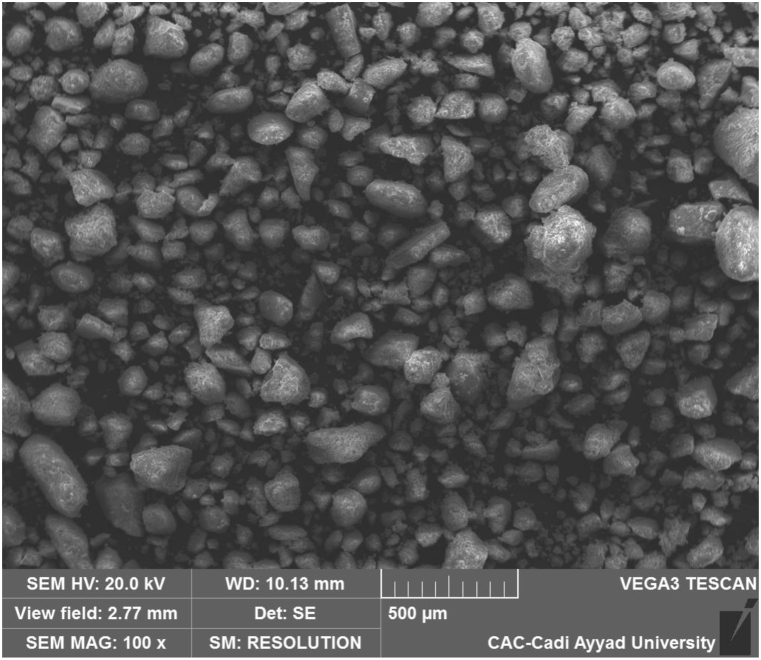
SEM micrograph of dust particles.

Fig. 5 presents SEM image characterization of the dust sample shows that the mineral content appears to be opaque, translucent, and semi-translucent, and this feature may cause light attenuation. The mineral density was high, which can slow down the flow intensity and cause light absorption. Most of the particles are small, dense, and spherical, which can result in light attenuation.

### 3.2 Summary of weather data

**Fig. 6:**
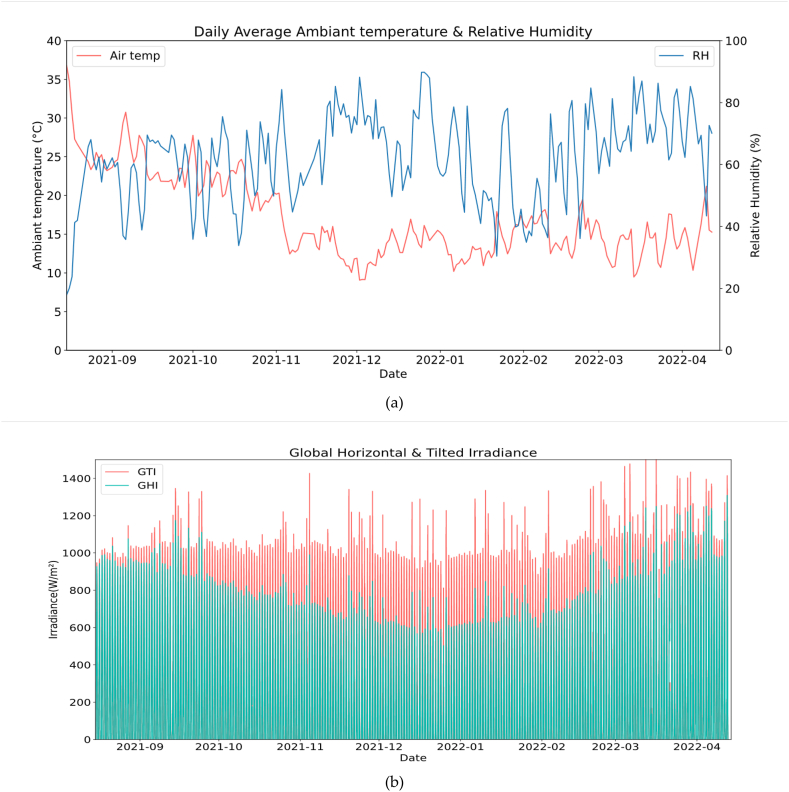

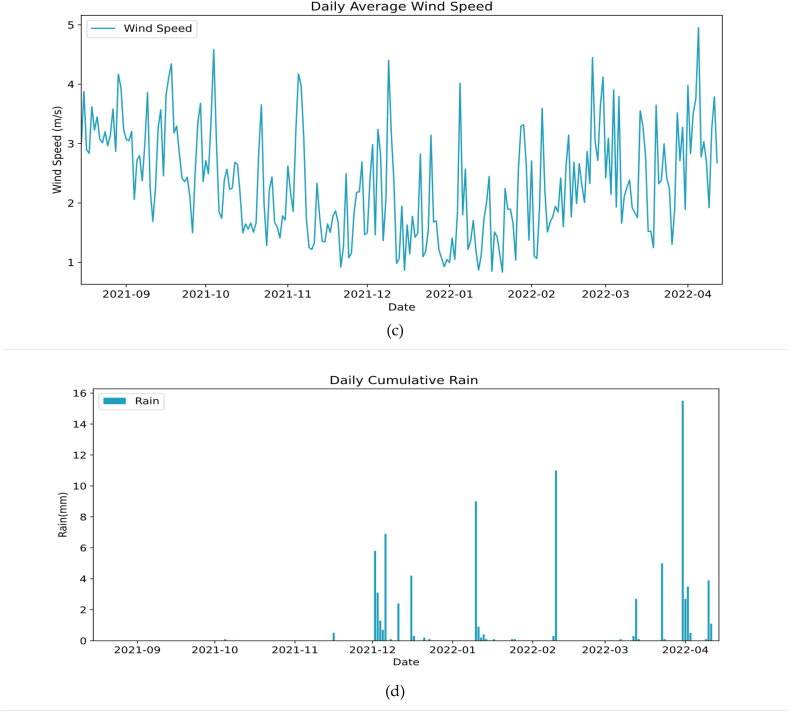
Meteorological parameters; (a) Ambient temperature & relative humidity; (b) Wind speed; (c) Global Horizontal & Plane of array irradiance; (d) Rain events.

Fig. 6a represents the evolution of air temperature and relative humidity over time. The evaluated weather data starts from august 12, 2021 to April 10, 2022, which corresponds to 8 months of exposure time during which PV systems will be evaluated. We remark that this period of exposure is divided into two main periods P1 [From August 15 to November 1] represent the dry period where high ambient temperatures and low relative humidity occurs. For the ambient temperature, it ranges from a maximum of 36 °C and a minimum of 20 °C while relative humidity ranges from 17.91% to 75.45%. The second period P2 [From November 1 to April 10] represents the winter period where low ambient temperatures and high relative humidity occurs. The ambient temperature, it ranges between 9.15 °C and 21.19 °C while relative humidity ranges from 30.43% to 89.83%. Regarding Wind speed variability, during the test period, it has a daily average between 1 m/s and 5 m/s with relatively low values between December and February.

Otherwise, it can be observed in Fig. 6b that the GTI remains always greater than or equal to the GHI, which is normal due to the inclination of PV panels to the optimum tilt (32°) for the purpose of producing the maximum yield during the year. We can also remark that the GHI decreases compared to the GTI in the period between October and February, this is mainly due to the fact in the winter season the declination of sun is lower, so the GHI is lower and GTI keeps collecting maximum irradiance due to the chosen inclination of 32°.

Fig. 6c represents the wind speed variation during the period of the study which remained low and does not exceed the maximum value of 5 m/s. As shown in Fig. 6d the period of the test is considered one of the driest seasons in Benguerir Morocco, with a significantly low rain rate, with a maximum of 15 mm/day reached on March 14, 2022.

### 3.3 Water and time monitoring during the cleaning period

The cleaning protocol is performed using a pressure sprayer machine and a brush to remove the accumulated dirt on top of the PV modules. Solar panels will be cleaned in either the morning or the evening time. Time and amount of water will be monitored during each cleaning cycle (Day 15, Day 30) to evaluate the easiness, fastness, and efficiency of the dirt cleaning in each situation. The graph below shows the amount of water and time spent during the first two cleaning cycles.

**Fig. 7:**
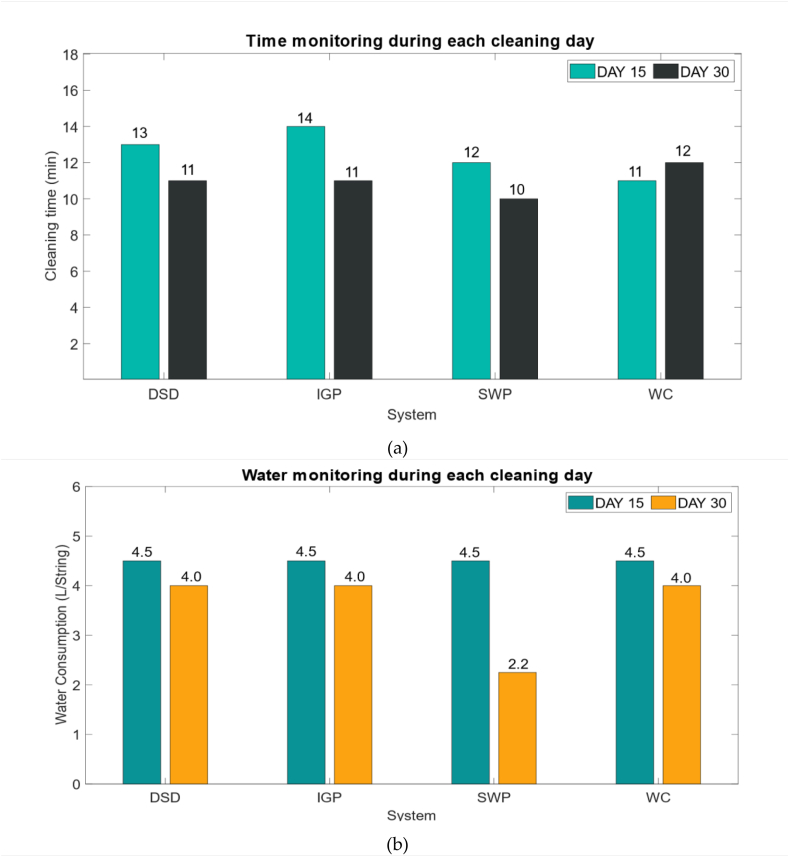
(a) Water consumption monitoring for each string; (b) Cleaning time monitoring for each string.

It can be observed in Fig. 7 a that after 15 days of outdoor exposure the application of the SWP solution during day 30 reduces the amount of water used by 50%, in comparison with the Water Cleaned (WC) string during day 15. Also, the cleaning time as shown in Fig. 7 b has been decreased from 12 min (cleaning with water) to 10 min (cleaning with SWP solution), nevertheless for the second period after 30 days of exposure it has shown only −0.5 L of water saved compared to the other solutions. Another analysis that can be observed regarding the “Water Cleaned” is that cleaning with only water makes the panels' surfaces hard to clean which confirms the increase in the duration of cleaning. The other strings coated with IGP and DSD show the same behavior by reducing the amount of water from 4.5 [L/string] during day 15 to 4 [L/string] during day 30, which reflects the effectiveness and easiness of dirt cleaning using CHEMITEK's products.

### 3.4 I–V curve tracer results

**Fig. 8:**
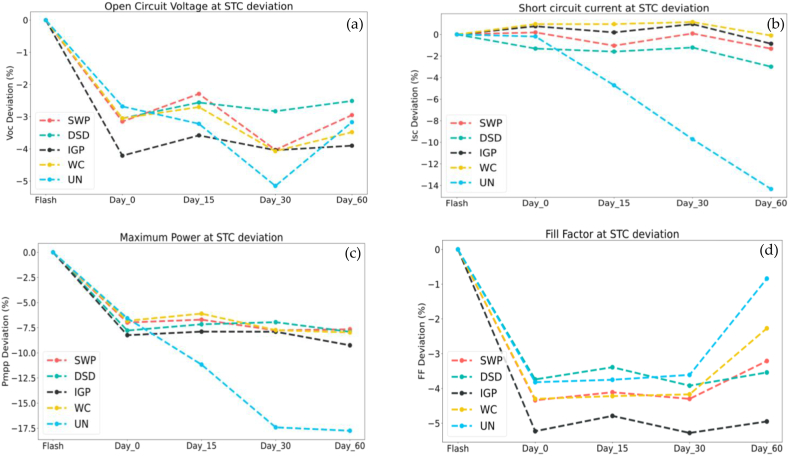
I–V tracer experiments results: (a) Open circuit voltage deviation; (b) Short circuit current deviation; (a) Maximum power point deviation; (a) Fill factor deviation.

The results obtained for the tested modules samples in the flash test and after the cleaning events are corrected to STC following the IEC 60891 standards and revealed the following results in Fig. 8 below.

1In Power output, a significant decline in the uncleaned string that reached −17.5% after day 60 of exposure without cleaning while DSD and SWP performed better than WC and IGP respectively.
2The uncleaned Short-circuit had the most notable reduction −14% related to dust accumulation, while there are no significant variations in Voc in the sample modules, being slightly higher in DSD.
3Since the sample modules are not perfectly identical, the slight changes in module measurements between cleaning events are attributed to module mismatching.

### 3.5 Performance indicators for grid connected PV systems

#### 3.5.1 Monthly energy yield

**Fig. 9:**
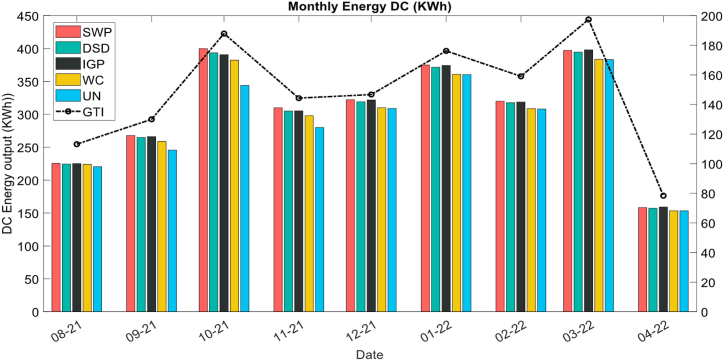
Monthly energy DC.

Fig. 9 represents the evolution of the monthly energy yield (kWh) (DC side) and the monthly global in plane irradiance GTI (W/m^2^) over time during the test period where the 5 PV systems (strings) are evaluated and taking into consideration the effect of cleaning that occurs in the 26 August (Day_15), 10 September (Day_30), 10 October (Day_60) and November 10. The system's energy output in the first cleaning cycle (August) is relatively identical, the differences occur after the successive cleaning, where the SWP was the highest-performing string during the dry season. The energy output reaches its maximum in October due to the high reference yield. During the last period of the study and with the number of rain rates measured, the IGP outperforms the SWP and DSD respectively during days of precipitation. In general, the washing solution string (SWP) and the coated strings (IGP, DSD) outperform the reference strings (WC, UN) in all the periods.

#### 3.5.2 Monthly array yield

**Fig. 10:**
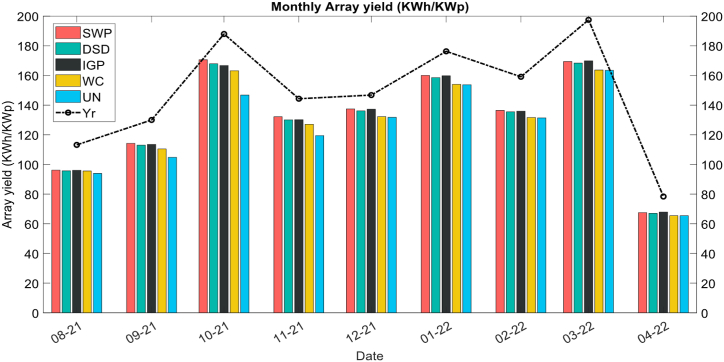
Monthly array yield.

Fig. 10 represents the evolution of the daily Array yield (kWh/KWp) (DC side) over time computed using Eq. (4). It can be observed that during the month of august SWP performs slightly better than the hydrophobic coating systems due to the application of the SWP solution during Day 0 (First cleaning), and this effect can be observed during the dry season. The same behavior is observed during September, where SWP systems outperform the IGP and DSD, whereas the uncleaned system's yield dropped down due to the non-cleaning followed by the water-cleaned system which remains below the IGP and DSD. After the last cleaning on November 10, it can be observed that the SWP has the highest output between November and February with very low rain rates, while in the period between March 10 and April 10, the IGP outperforms the SWP solution which can be due to the higher rain rates.

#### 3.5.3 DC capture losses

**Fig. 11:**
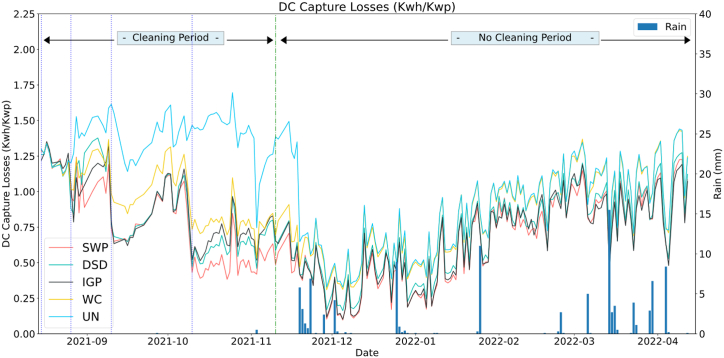
DC capture losses.

Fig. 11 represents the evolution of the array losses (computed using Eq. (4)) that occur in the system (DC side) over the test period. It can be observed that the systems behave the same way until the second cleaning day (Day_15), where PV system capture losses increase for the UN string due to dust accumulation. The DSD and IGP system losses have dropped below all other PV strings right after the second cleaning (Day_15) which is a good sign of the effectiveness and the easiness of the dust removal using only water cleaning. After the cleaning of day 30 and within the 30-day cleaning cycles (10 Sep – 10 Nov) the SWP has the lowest capture losses followed by IGP and DSD respectively, while the UN remains with the highest losses due to dust accumulation. While with the rain events occurring in 19th-23rd November the UN regains its performance and becomes relatively identical to WC, the SWP remains the highest performing with the lowest losses due to the low rain rates. With the increasing rain rates, the IGP outperforms the SWP in the last period of the test (March–April).

#### 3.5.4 Performance ratio PR

**Fig. 12:**
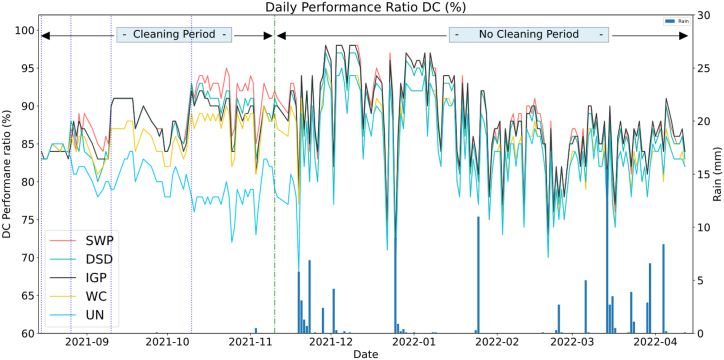
Performance ratio DC.

As illustrated in Fig. 12 below, the obtained PR values computed using Eq. (3) showed that.

1After the second cleaning (Day_15) [26 Aug–Sep10], the performance ratio of the “Uncleaned” string remains below all others PV string's PR while all other string's PR increased during this day due to dust removal. It is clearly observed that PV strings using coatings (SWP, IGP, and DSD) perform better during the cleaning days where their PR is the highest compared to the Water cleaned string.
2After Day_30 [Sep10 – Oct 10], the SWP remains the highest performing string followed by IGP and DSD respectively. In the second 30-day cycle [Oct 10 – Nov 10], the SWP surpasses the other systems, while DSD outperforms the IGP in the first days after the cleaning.
3Following the last cleaning applied on November 10 [Nov 10 – Feb 10], the SWP remains the highest-performing system due to the effectiveness of the solution in the low rain rates occurring during this period, while the IGP regains its performance after the first rain events occurring in 19th-23rd November. The UN becomes very close to the WC after the rain events and has the same behavior.
4During the last period [Feb 10 – April 10], at first, the SWP remained the highest performing string with very low rain rates, although with the increasing rain rates after March 6th, the IGP surpassed the SWP, whereas the DSD remained below SWP.

#### 3.5.5 Cumulative energy gain

**Fig. 13:**
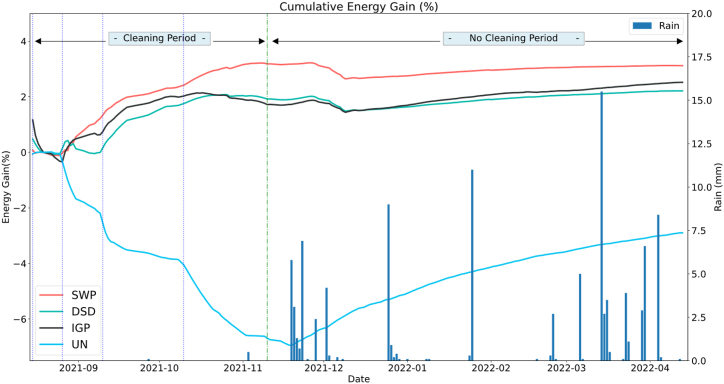
Cumulative Energy gain.

Fig. 13 represents the energy gain accumulated by each system compared to the water cleaned system (Calculated using Eq. (6)). As shown in the graph above, all systems have approximately the same energy generation. Otherwise, the SWP system is increasingly deviating to reach more than 3.22% of cumulated energy gain compared with the Water cleaned in the dry season, followed by the DSD and IGP reaching 2.01% and 1.89% respectively of the cumulated energy gain, while the Uncleaned system starts to lose its energy with a negative energy gain from the Water cleaned system, reaching a value of −6.94% at the end of the dry period. Otherwise, during the rainy period of the test, the gain of the systems has dropped due to the natural cleaning of the WC string and the uncleaned system regains its performance, during the period between Nov 10 - Apr 10, while the IGP surpasses the DSD and reach a gain of 2.52% and 2.21% respectively while SWP has the highest gain of 3.12% which is due to the low rain rates measured during the period of the test.

#### 3.5.6 Performance ratio deviation compared to soiled reference system

To study the behavior of the different coatings and cleaning solutions in dusty environment, a confrontation of performance ratios with the uncleaned PV system was conducted to identify how effective these solutions are in dusty environments. the deviation is calculated using the following equation:

(7) $${dev}_{sys}=\left(\frac{PRsys}{PRun}-1\right)*\;100$$

**Fig. 14:**
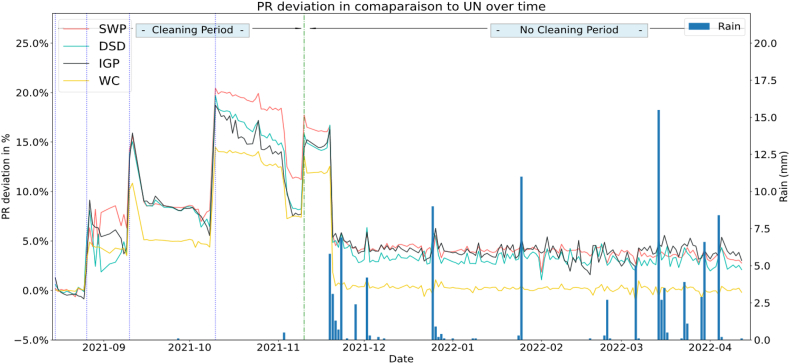
Performance ratio deviation compared to reference uncleaned system.

Fig. 14 represents the deviations of PR for each PV string using as a reference the Uncleaned String. We can conclude that PV strings start to deviate from the uncleaned right after the first cleaning day (26 August) with the highest rate maintained by the SWP system. While after the second cleaning (10 September), we can see that DSD, IGP and SWP deviate with the same rate from the uncleaned system, and that the Water cleaned system keeps its deviation lower and constant at approx. 5% from the Uncleaned system. The deviation reaches its maximum value of 20.46% after the cleaning of day_60 (October 10), This behavior can reflect the advantage of the coating solutions used during this period of test. After the rain events occurred in 19–23 November the uncleaned system regained its performance and the gain dropped to an average value of 3% for the 3 solutions with the same behavior stated before in the Performance ratio graph, while during the cleaning and rainy days, the DSD and IGP outperform the SWP.

## 4 Discussion on the value of cleaning solutions

The field test has been conducted for evaluating multiple types of cleaning solutions on a grid-connected PV system divided into 5 sub-systems (Uncleaned, Water Cleaned, Solar Wash Protect, Industrial Glass Protect, and D-Solar Defendor), each system is composed of 7 modules in series, with a rated power of 2.345 kWp. The PV system is in the solar test field at the Green Energy Park in the city of Benguerir, Morocco. The system's performance is evaluated during a period of 8 months for the period between August 2021 and April 2022, characterizing the dry and rainy seasons and using a particular cleaning protocol.

The three main systems (SWP, IGP, DSD) are compared with respect to the uncleaned system, which remain without any cleaning during all the period of the test, and the Water cleaned which was cleaned with only water, in order to evaluate the hydrophobic coatings and cleaning solutions as a first result during the cleaning period, SWP shows a reduction of 50% in terms of water consumption with respect to the water cleaned system. A reduction of time is also observed during the cleaning period with IGP and DSD, while for the Water cleaned string, cleaning with only water makes the panels’ surfaces harder to clean.

Concerning the performance evaluation of the PV strings, it was necessary to see the electrical behavior of the tested systems under outdoor conditions. During this study and for each string, we have been analyzing their production mainly the DC energy production, Array yield, Array capture losses. In addition, to conclude on their performance some key performance indicators have been set, mainly the Performance ratio and the cumulative energy gain in order to study the performance of the different anti-soiling solutions and to identify which is the most effective one in a dusty environment.

The analysis of the KPI demonstrated that during the first two weeks, the behavior of all PV strings was approximately the same. After performing the second cleaning (which corresponds to Day_15 of the cleaning protocol), the “Uncleaned” performance and production are below all other PV strings due to dust accumulation, while the systems with the coatings (SWP, IGP, and DSD) represent a higher performance (Higher PR and higher energy generation) compared to the Water cleaned system. During the period of cleaning, either IGP, DSD, and SWP represent good behavior in terms of dust removal, where their losses become the lowest compared to the water cleaning system. During the cleaning period [Aug 12th – April 10th] corresponding to the dry season the SWP remains the highest performance system reaching a gain of 20.46% with respect to the uncleaned system after the cleaning of Day_30 followed by DSD and IGP reaching a gain of 19.71% and 18.75% respectively. With the increasing rain rates starting from November 19th the IGP surpasses the DSD while WC becomes identical to the Uncleaned. During the period of November–February SWP remains the highest performing string due to the effectiveness of the cleaning solution and the antistatic coating left behind on the surface of the panel lasting up to 6 months, and the relatively low rain rates, although during March and April with the high rain rates the IGP surpasses the SWP system reaching an average gain of 3.7% compared to WC (SWP reaches 3.27% and 2.58% for DSD).

To conclude on the anti-soiling solutions and their performances, SWP is more performant in terms of dust removal when applying the SWP solution during the dry season (August–February) with low rain rates, while during the rainy period (March–April) the IGP is more performant than SWP followed by DSD with a small difference.

## 5 Conclusion

This study examines the use of two innovative hydrophobic coatings and cleaning methods to 5 strings composing a 11.72 kWp monocrystalline PV systems under real exposure conditions. The testing was conducted in the semi-arid climate of the Moroccan city of Ben Guerir.

The findings of the research that was carried out allow one to draw the following conclusions.

✓ The dust that settles on the ground in and around Benguerir City is a significant contributor to the loss in power that comes from solar PV modules.
✓ The application of coatings and cleaning products as a method for reducing dust is a useful approach in the process of cleaning photovoltaic panels.
✓ When compared to the water-cleaned system, the losses sustained by INDUSTRIAL GLASS PROTECT (IGP), D-Solar Defendor (DSD), and Solar Wash Protect (SWP) throughout the cleaning process are significantly lower, indicating that these processes are performing excellently in terms of the removal of dust.
✓ After 9 months of operation, the coated PV panels improve 10% efficiency over the reference system in the first 3 months (cleaning time). After 6 months, the non-cleaning time gains 5% efficiency. During outdoor exposure, coated systems gain 3% more energy than water-cleaned systems.
✓ The Solar Wash Protect (SWP) has demonstrated to be the most efficient method of action in comparison to the other solutions for the semi-arid climatic conditions that prevail in the city of Ben Guerir.

This paper is part of a larger series of future research that will be conducted, all of which will focus on the topic of soiling mitigation techniques and analyze them in greater depth. For example, the investigation and evaluation of innovative passive cleaning solutions, such as hydrophilic, super hydrophilic, and superhydrophobic coatings, under varying climatic conditions and possibly with other PV technologies.

## Author contribution statement

Abderrazzak Elamim; Said Elhamaoui: Conceived and designed the experiments; Performed the experiments; Analyzed and interpreted the data; Contributed reagents, materials ,analysis tools or data ;Wrote the paper.

Khalil Tijani; Performed the experiments; Contributed reagents, materials tools or data ; Wrote the paper.

Abouabakr Benazzouz; Cesar Martins; Bruno Queiroz; Clara Faria: Analyzed and interpreted the data;Wrote the paper.

Abdelattif Ghennioui: Contributed reagents, materials, analysis tools or data.

## Data availability statement

The data that has been used is confidential.

## Declaration of competing interest

The authors declare that they have no known competing financial interests or personal relationships that could have appeared to influence the work reported in this paper.
